# Scoping Review on the Impact of Outbreaks on Sexual and Reproductive Health Services: Proposed Frameworks for Pre-, Intra-, and Postoutbreak Situations

**DOI:** 10.1155/2021/9989478

**Published:** 2021-09-08

**Authors:** Syed Khurram Azmat, Moazzam Ali, Fahad Javaid Siddiqui, Syed Farhan Ali Tirmizi, James Kiarie

**Affiliations:** ^1^Marie Stopes Society, Karachi, Sindh, Pakistan; ^2^AAPNA-Institute of Public Health, Jinnah Sindh Medical University, Karachi, Sindh, Pakistan; ^3^UNDP-UNFPA-UNICEF-WHO-World Bank Special Programme of Research, Development and Research Training in Human Reproduction (HRP), Department of Sexual and Reproductive Health and Research, World Health Organization, 20 Avenue Appia, 1211 Geneva, Switzerland; ^4^Pre-hospital & Emergency Research Centre, Duke-NUS Medical School, 8 College Road, 168579, Singapore; ^5^Public Health Surveillance and Infrastructure (PHSI), Alberta Health Services, Alberta, Canada

## Abstract

**Introduction:**

Recent experiences from global outbreaks have highlighted the severe disruptions in sexual and reproductive health services that expose women and girls to preventable health risks. Yet, to date, there is no review studying the possible impact of outbreaks on sexual and reproductive health (SRH). *Methodology*. Studies reporting outbreaks impacting sexual and reproductive health and pregnancy outcomes were identified using MEDLINE, Embase, and ISI-WoS. Reported impacts were reviewed at systems, community, and legislative levels.

**Results:**

The initial run listed 4423 studies; the 37 studies that met all inclusion criteria were mainly from Latin America and Africa. Studies on outbreaks of diseases like Zika and Ebola have documented declines in facility-based deliveries, contraceptive use, and antenatal and institutional care due to burdened healthcare system. Service usage was also impacted by a lack of trust in the healthcare system and system shocks, including workforce capacity and availability. At the community level, poverty and lack of awareness were critical contributors to poor access to SRH services. Assessing the target population's knowledge, attitude, beliefs, and behavior and using health literacy principles for communication were fundamental for designing service delivery. Online resources for SRH services were an acceptable medium of information among young adults. In outbreak situations, SRH and pregnancy outcomes were improved by implementing laboratory surveillance, free-of-cost contraceptive services, improved screening through professional training, and quality of care. In addition, mobile health clinics were reported to be effective in remote areas. *Knowledge Contribution*. In outbreaks, the interventions are categorized into preoutbreak, during, and postoutbreak periods. The proposed steps can help to improve and do course correction in emergencies. Though conducted before the COVID-19 crisis, the authors believe that lessons can be drawn from the paper to understand and mitigate the impact of the pandemic on sexual and reproductive health services.

## 1. Introduction

In low-resource settings, disease outbreaks have the potential to make health service delivery a challenge. Outbreaks tend to put enormous pressure on the health system to deliver quality healthcare [1]. Poor healthcare quality has been shown to adversely affect health outcomes, especially in low- and middle-income countries [1]. Furthermore, during the outbreak, stressed health systems can be overwhelmed in efforts to provide healthcare to their respective populations due to an increase in demand. In such situations, vulnerable and at-risk population groups such as pregnant mothers and children are especially at greater risk of having adverse health outcomes [2]. It is, therefore, imperative that the impact of outbreaks on sexual and reproductive health (SRH) and pregnancy-related outcomes of vulnerable populations in low-resource settings be further studied for improved response.

Outbreaks are characterized by a localized increase in the number of cases of a particular disease in comparator timeframe [3]. Various social and environmental factors, including overcrowding, armed conflicts, and global warming, drive outbreaks [4, 5]. Outbreaks have the potential to impact SRH and pregnancy-related outcomes [6–10].

Data from 219 countries shows that there has been a significant global rise in outbreaks of human infectious diseases. According to a study, from 1980 to 2013, the number of outbreaks and emerging infectious diseases has increased [9].

Besides straining healthcare delivery, outbreaks also result in considerable morbidity and mortality, instill widespread fear and social disruptions, and attract large-scale publicity due to their overall damaging effects [1]. For example, during Ebola outbreaks, assessments from West Africa in 2014 showed that lapses in service delivery resulted in the collapse of public trust in health systems [1]. In addition, health system response during outbreaks may be hampered by a low density of human resources for health, low capacity for disease surveillance in the community, infrastructural deficits in health facilities, and weak supply chains for essential medicines [11].

Adequate outbreak response entails establishing strong linkages between health system resilience, quality of care, and global health security [1]. During outbreaks, the health system challenges can exacerbate health inequalities confronted by at-risk populations not limited to pregnant women and children but for women, girls, adolescents, migrants, refugees, and the poor. Adverse sexual reproductive health and pregnancy outcomes have been observed postsyphilis, Zika, Ebola, and measles infection outbreaks [1, 2, 12]. For example, an impact evaluation of the Ebola outbreak on health systems and population health in Sierra Leone showed a postoutbreak surge in teenage pregnancies. The same study also recorded a reduction in vaccination coverage, highlighting the consequences of outbreaks on fragile health systems, especially in developing countries. Additionally, Zika virus infection is associated with adverse reproductive outcomes that include miscarriage, fetal demise, stillbirth, and congenital malformations such as microcephaly and intrauterine growth restriction [13].

There is a potential of aggravation in health inequalities confronted by vulnerable segments of the population, such as pregnant women in outbreaks. While Zika infections are associated with adverse reproductive outcomes, it has been observed that even in such situations, the abortion policies remained essentially unchanged [13]. A scoping review of Zika virus literature identified the need for gathering more evidence to improve the understanding of the Zika virus and its impact on public health [14, 15].

### 1.1. Rationale and Objectives

To inform policy guidance and future planning, there is a need to analyze the literature and evaluate the impact of outbreaks on SRH and pregnancy outcomes. To date, we have not come across any systematic literature review that addresses this topic. This scoping review examines existing evidence on how disease outbreaks lead to adverse SRH outcomes directly or indirectly by impacting health systems, community, and policy response. In addition, we report lessons learned from effective interventions to reduce adverse SRH outcomes before, during, and postoutbreaks and their implications for policymakers.

## 2. Methodology

**Figure 1:**
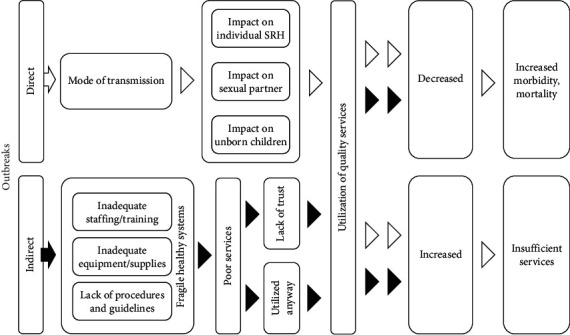
Conceptual model: description.

This scoping review is conducted using Arksey and O'Malley's methodology and Levac's methodological enhancement [16, 17]. In addition, the present scoping review has adopted a systematic approach for mapping evidence to identify main concepts on how outbreaks lead to adverse SRH outcomes directly or by impacting health systems, community, and policy response [12]. For the description of the conceptual model used in this scoping review, kindly refer to Figure 1.

### 2.1. Impact on SRH

- Direct effect
- Indirect effect: depending on the nature of disease and transmission mode, outbreaks affect individual SRH through impacting access and utilization of SRH products and services. This, in turn, through an inadequate health system response, leads to an increase in morbidity and mortality

The burden on a fragile health system increases after an outbreak. Lack of appropriate procedures and guidelines leads to system disruption and collapse, resulting in fewer services (preventive/curative) for patients causing an increase in morbidity and mortality.

### 2.2. Review Question and Scope

How do outbreaks directly lead to adverse SRH outcomes impacting health systems, community, and policy response? In addition, kindly refer to Table 1 for the key definitions used in this scoping review for developing key concepts.

### 2.3. Search Strategy

Studies/literature relevant for this scoping review were identified by searching the following electronic databases of the published literature: MEDLINE, Embase, and ISI-WoS (the appendix). These databases cover most of the published peer review on outbreaks and SRH; both being intensely studied topics are unlikely to remain unpublished. Considering the 1994 International Population and Development Conference held in Cairo as a landmark for SRH, we limited our search from January 1995 to August 2018 [23]. Only English articles were considered for this scoping review. The team leader experienced in the literature search in consultation with a librarian identified a draft list of search terms. The WHO technical lead discussed and approved the final set of search terms before running the search. All the records identified through different sources were downloaded in the EndNote library and were screened by two individuals as part of the research team. Studies that report outbreaks having an impact on SRH and pregnancy outcomes were identified.

### 2.4. Relevance Screening and Inclusion Criteria

Based on the approved protocol, this scoping review consisted of three levels of screening: duplicate removal, title and abstract review, and full-text review.

A screening form was developed before screen abstracts, titles, and keywords of identified citations. EndNote “Find Duplicate” tool was also used to remove duplicates, using author, year title, and volume field matching. A rerun was done by the team leader experienced in literature screening. An eligibility criterion was applied less stringently at this stage to avoid losing any relevant record. Any article that is deemed appropriate for the question of interest is included in the full-text screening stage. In the third level, the team established whether the full-text articles meet the inclusion/exclusion criteria through consultation. Hard to categorize articles were referred to another team member who reviewed and shared his opinion with the team leader, and the decision was made with consensus. An article was of relevance if (a) it is reporting an outbreak (b) concerned with sexual reproductive health and pregnancy outcomes at title abstract/abstract stage. At the full-text screening stage, the article should also be reporting on (c) healthcare service delivery or quality, or (d) access or utilization to healthcare, or (e) health-seeking behavior. The criteria were tested on a sample of abstracts before beginning the abstract review to ensure that they are robust enough to capture any articles with evidence on outbreaks and conditions of interest. Articles from high-income countries (World Bank Classification, World Bank Country and Lending Groups: Country Classification. Available from URL: https://blogs.worldbank.org/opendata/new-country-classifications-income-level-2018-2019, last accessed: Sep 09, 2018.) were excluded as the healthcare systems are often strong enough to bear shocks of outbreaks and do not represent the challenges faced by low- and middle-income countries.

### 2.5. Study Characterization and Extraction

As described, the team leader developed a data collection instrument to confirm study relevance and extract study characteristics. In addition, the form was pretested by the WHO technical lead before implementation to ensure the data collection instrument is capturing the information accurately.

### 2.6. Scoping Review Management, Data Charting, and Analysis

The team leader did data abstraction; however, 5% of randomly chosen extracted forms were done by another team member. No significant discrepancies were found, and the data was compiled in a single literature review software program and synthesis and was downloaded into a single Excel spreadsheet in Microsoft Excel software for validation and coding. Following this, the identified quality indicators were extracted and coded.

### 2.7. Study Contribution

There is a considerable shortage of high-quality published data related to SRH and pregnancies in outbreaks, and evidence is scarce about their background in humanitarian settings. Obstacles include access, cultural and ideological barriers, data challenges, financial and resource constraints, and systemic and sectoral challenges. Higher-quality evidence on SRH and pregnancy-related services in outbreaks through a collective framework is needed to improve access and services. This information is essential for policymakers, donors, and program managers working within SRH in pandemics and outbreaks to making informed decisions at national and global levels.

### 2.8. Dissemination and Ethics

The results from this scoping review will guide the next phase of a multifaceted research program that will set the direction of further research in the short term and ultimately the development of guidelines that can be implemented at the system level to measure and monitor SRH and pregnancy outcomes during outbreaks. This study did not require ethics approval as it is reviewing data from publicly available materials.

### 2.9. Limitations

Single-person screening might not be the best way to select studies; however, having an experienced author taking a sensitive approach towards inclusion rather than exclusion reduced possible bias. Excluding non-English language articles would be a major limitation as we might have missed research published in Spanish and French. Nevertheless, if major themes are captured, our objective to guide the future course of action is primarily achieved.

## 3. Results

### 3.1. Scoping Review's Descriptive Statistics

**Figure 2:**
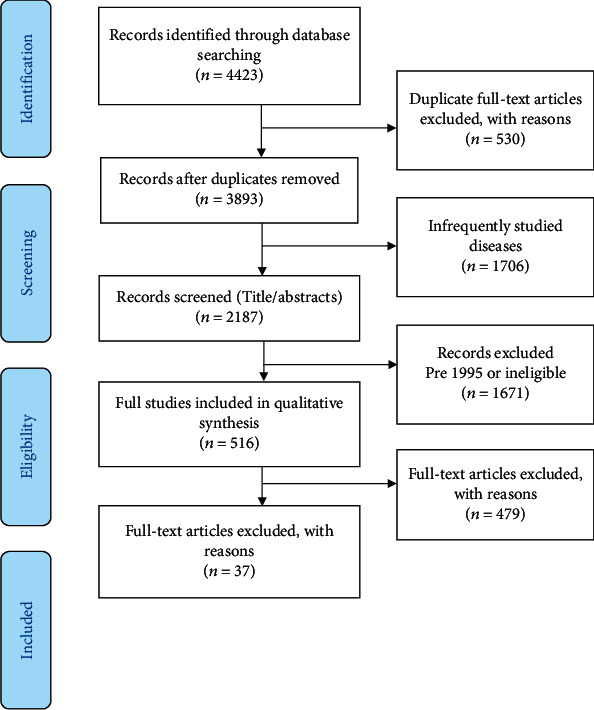
PRISMA flow chart.

Using the PRISMA guidelines (as much as applicable for the scoping review), our initial run of the search strategy based on all outbreaks on the WHO webpage list returned 4423 records. After removing 530 duplicate records and 1706 irrelevant diseases (refer to Figure 2), we were left with 2187 records to be screened at the title abstract stage. After removing pre-1995 and ineligible records at the title abstract step, we retrieved full text for 516 records. Out of these, 37 met inclusion criteria and were included in the analysis. Most of the articles were published between 2017 and 2018, with only one in the late 90s. Most of the literature was from Latin America and Africa, with only a handful from Asian countries.

Influenza, Ebola, and Zika were the most frequently studied diseases, followed by meningitis, SARS, and cholera (refer to Table 2).

Most of the studies conducted were at the subnational level; however, studies on national levels were also performed (refer to Tables 3 and 4). Study designs adopted were prospective, retrospective, and cross-sectional. Most studies either focused on SRH or pregnancy outcomes with a few exceptions.

### 3.2. Thematic Analysis

#### 3.2.1. Health-Related Knowledge, Attitude, and Behavior

Contraceptive commodity sales have been used to indicate healthcare behavior in certain epidemics [24–26]. This is primarily the case with Zika outbreak studies. Therefore, authors have investigated changes in the sales of contraceptives at the country level and pre-, during, and postoutbreak sales. Not much variation has been observed, but very low access to long-acting reversible contraception has been noted [27].

At subnational-level studies, some have found improved access to and utilization for contraceptive services and improved uptake of most effective methods when provided free of cost [28]. For example, during Zika outbreaks in Brazil, most women wanted to delay pregnancies, but only women with high SES control their intentions [29]. In another study from Brazil, low awareness about contraception was observed, with few women considering a change in pregnancy intentions due to the outbreak [30]. A study on people with disabilities also found low levels of awareness about contraception in South America [31].

#### 3.2.2. Health System Capacity Building or Readiness

In Brazil, the critical elements to expanding services in SRH and to tackle an outbreak were identified as contraceptive availability, method mix, and utilization pattern which were used as measures of readiness of the health system [32, 33]. During an outbreak, emergency contraceptives were available in almost all pharmacies staffed by pharmacists with a low level of knowledge [34]. Likewise, in Puerto Rico that reported high unintended pregnancies, investigators found a significant unmet need for contraceptives during an outbreak where a large proportion of pregnancies were unintended [35]. A study conducted in an emergency medicine department in Brazil found that a double epidemic of dengue and Zika disrupted routine care [36]. A multicountry study found that the upgradation of ultrasonography services by training for equipment care resulted in improved quality of care [37].

#### 3.2.3. The Burden of Disease Measurement

Studies assessed the load on health systems in different ways. For example, lab-based surveillance was implemented in Haiti to measure Zika virus disease cases [38], while in several asymptomatic cases, pregnancy outcomes in Zika and Ebola-infected mothers and trends of incident Zika/Ebola cases have been reported to assess burden on various aspects of the healthcare system [39–41].

#### 3.2.4. Healthcare Service Utilization

Ebola impacted the trust in the healthcare system to provide quality services during outbreaks. Therefore, the most frequently studied aspect was a decline in service utilization. A national-level study for rural settings in Guinea reported a decrease in contraceptive use, antenatal care visits, and institutional deliveries during the Ebola outbreak [26]. However, it also reported postoutbreak recovery of contraception service utilization but not the other two. In several studies, utilization of institutional care, facility-based deliveries, and delay in antenatal visits have been assessed and found to indicate a decline in utilization throughout with little or no recovery in Guinea, Liberia, and Sierra Leone [42–48]. Sierra Leone found that lack of friendly attitude in health facilities and flawed referral system were barriers to facility-based delivery [49].

#### 3.2.5. Healthcare Service Provision

Triage and isolation protocol implementation and mobile health clinics were found to be effective in Sierra Leone [50, 51]. In addition, despite being at a very high risk of contracting Ebola during the outbreak, nurses and midwives continued to provide care in Sierra Leone due to various motivations, including professional duty, responsibility to the community, and religious beliefs [52].

#### 3.2.6. The Burden of Disease

A few other studies reported the burden on the disease to indicate the insufficiency or low quality of healthcare service in the wake of an outbreak where lives could have been saved if not the case [53–55]. In Sierra Leone, MNCH services declined due to a weak system, confusion over policy, and the magnitude of the outbreak. Services by CHWs improved with clear instructions despite mistrust by the community. CHWs remained more effective than outsiders [56].

A study from Turkey reported reduced adverse outcomes by implementing various strategies to handle the pregnant patient load. Strategies included admittance of suspected and confirmed cases to the hospital and testing for potential H1N1 infection [57]. Another study from Brazil observed that pregnant women with influenza consumed a higher amount of healthcare services than their noninfected counterparts, possibly due to a higher death rate [58].

A study from Haiti reported improved outcomes in low-resource settings among pregnant women with cholera through a women-centered approach of providing obstetric and neonatal services and supervision of hydration status [59].

A study from Sri Lanka reported an outbreak of fungal meningitis after using improperly stored donated medical supplies during pregnancy and delivery care (including anesthesia medication for cesarean section) after the tsunami highlighting the fact that not only provision of supplies is essential, but these need to be appropriately handled until used [60].

## 4. Discussion

Scoping reviews are used to map out key concepts behind an area of research. This scoping review analytically reviews the available literature on the impact of outbreaks on SRH and pregnancy outcomes. It reports on the impact of outbreaks on SRH outcomes, whether direct or indirect, affecting health systems, any possible consequences to the community, or policy response. Also reported are the lessons learned from effective interventions aimed at reducing adverse SRH outcomes. The intention is to collate evidence to guide future policy and planning. It is documented that outbreaks are associated with undesirable reproductive health outcomes with no impact on relevant policies, e.g., Zika and Ebola outbreaks [13]. At the time of this review, there was no existing scoping/literature review encompassing this topic. The major infectious diseases causing outbreaks from the pool of the selected studies included influenza (37%), EVD (15%), meningitis (9%), Zika (11%), cholera (6%), and SARS (6%) indicating a range of diseases that can impact SRH among vulnerable populations (Table 3). These diseases have affected the local and global populations and health systems differently.

We report the findings of this review on health system impact in terms of (i) supply-side impact, i.e., services, human resources, and policies; (ii) demand-side impacts, i.e., utilization pattern of SRH services; and (iii) enabling environment, i.e., legislation and policy implications levels.

### 4.1. Supply-Side Impacts

Exploration of supply-side issues within situational and environmental determinants such as availability and access to modern contraception, vector control, and sociocultural factors related to pregnancy and family planning to prevent diseases like Zika and reproductive health has been emphasized [61].

The burden on the healthcare system is a primary concern during any outbreak, especially in countries with limited resources. Different studies documenting Zika and Ebola outbreaks resulted in the decline in utilization of services [39–41, 53, 54]. Studies from West African countries like Sierra Leone, Guinea, and Liberia reported poor utilization of institutional care, facility-based deliveries, contraceptive use, and antenatal care during the Ebola outbreak due to overburdened healthcare systems [26, 42–48]. Similarly, inappropriate handling and storage of medical supplies that included anesthesia medication for pregnancy and delivery care, including cesarean surgery, were highlighted in a Sri Lankan study where an outbreak of fungal meningitis among pregnant women was reported posttsunami due to usage of improperly stored medicines [60].

During the Zika outbreak, few studies reported shortage and stockout of supplies, including contraceptives, availability of trained workforce, method mix, consumption, and access to long-term reversible contraceptive methods [27, 32]. During the Zika outbreak, free-of-cost availability of effective contraception resulted in better use [28]. The knowledge and use were also found low in emergency contraception pills, but they were available in most pharmacies [34]. However, Puerto Rico reported a significant unmet need for contraception, and a considerable proportion of pregnancies were unintended [35].

Innovative approaches to address the increase in service demands such as triaging patients, implementation of isolation protocols, and use of mobile clinics were effective in Sierra Leone. Personal attributes, including religious beliefs, professional duty, and sense of responsibility to the community of midwives and nurses, also played an essential role in keeping up healthcare services [52]. A study from Turkey reported reduced adverse outcomes due to influenza by implementing multiple strategies, including timely testing and treatment for H1N1 infection [57].

Another important aspect is that good-quality data on SRH in outbreaks were limited. As a result, there is a lack of evidence of their impact on accessibility and obstacles to using various contraceptive services [62]. Poor healthcare quality adversely affected health outcomes, especially in low- and middle-income countries [63, 64]. This was established in the Ebola outbreak in West Africa in 2014, during which gaps in service delivery combined with lack of public trust in health systems offered challenges to response and recovery [64].

### 4.2. Demand-Side Impacts

Reliability and trust in government organizations as a source of information have always been insufficient. This was elicited in a study of college students, which found low use of government websites compared to news media during the Ebola outbreak [65]. Likewise, the Ebola virus disease affected the trust in the healthcare system resulting in a decline in the utilization of services.

Healthcare service utilization was higher among pregnant women suffering from influenza in Brazil than nonpregnant women without the disease [58].

Few women considered changes in pregnancy intentions during the Zika outbreak due to low awareness regarding contraception in Brazil [30] and South America [31]. At the same time, there has been minimal variation in sales of contraceptives before, during, and after Zika outbreaks [27]. Other studies report improved access, uptake, and utilization of contraceptive methods at the subnational level [28]. Poverty and lack of awareness were the key determinants to poor utilization of reproductive health services. Sex education, access to contraceptives, safe motherhood, and safe abortion programs should be implemented to prevent discrimination [31]. Another study in Brazil during the Zika outbreak identified relatively low awareness and contraceptive knowledge as the main reason for fewer women changing pregnancy intentions [30].

Although previous research has shown online sources of information as an acceptable conduit among young adults [66], including sexual health [67], simple availability of information is not enough. An exploratory study assessing knowledge, attitude, belief, and behavior regarding reproductive health in the context of Zika reports difficulty in evaluating information and translating it into practice. For example, the behavior change was mainly related to mosquito control rather than the sexual transmission of the disease [68]. Thus, to promote understanding and use of information by the public, there is a need to integrate health literacy and communication principles in disseminating information [68, 69].

### 4.3. Enabling Environment Impacts

Important risk factors like gender have been identified as missing at the policy level. However, due to differences in exposure level, it needs to be considered during health planning [15]. Different studies have reported novel models to address the outbreak situations. For example, Haiti implemented a policy to strengthen laboratory surveillance to identify Zika cases and measure the burden [38]. A free-of-cost availability of effective contraception resulted in better uptake and utilization during the Zika outbreak in Puerto Rico [28]. During the EVD outbreak in Sierra Leone, policies to improve screening accuracy, nursing skills, IPC, and quality of care resulted in increased efficiency and quality of system depicted by the enhanced patient flow [50]. Similarly, the communities received mobile health clinics in Sierra Leone enthusiastically, particularly in remote areas [51].

Implementation of a surveillance system was also identified as an integral model which, through appropriate legislation, can be a valuable tool to counter outbreaks and their impact on SRH. For example, complex monitoring of Zika, including laboratories, vector control, social mobilization, and clinical care, was successful during the Zika outbreak in Haiti [33, 38]. Lack of availability of abortion guidelines also was noted in Brazil during the Zika outbreak [33]. Another study in Guatemala, El Salvador, Dominican Republic, Honduras, and Haiti emphasized the need for upgradation of diagnostic services in the context of the Zika virus epidemic [37]. In addition, there is a large gap between the demand and availability of contraception in Puerto Rico. In the backdrop of many unintended pregnancies, there was a need to improve availability and access to contraception through legislation or policy [35], which needs appropriate action from the highest level.

Renewed focus on global health protection has been demonstrated by establishing weaknesses in public health preparedness by outbreaks like Zika and Ebola [70]. The International Health Regulations (IHR) was updated in 2007, keeping outbreaks like Ebola and Zika. The aim was to prevent, protect, control, and provide public health response to the international spread of diseases. The broader scope of these regulations was to advocate for urgent action and strengthen national systems and infrastructure [71]. Quality FP services (known as QFP) were set as the standard of care for family planning to help reproductive-age men and women accomplish their desired spacing and healthy children [72].

There is significant evidence of vulnerable populations being at greater risk, primarily because of outbreaks like Zika, Ebola, and measles [73, 74]. Hence, there is a need to improve health literacy efforts that recognize risk perceptions as barriers to behavior change. Use of health literacy principles such as using local lay-man language should be part of such health communication messages targeted at establishing a connection between reproductive health and diseases like Zika and adopting the information for behavior change among reproductive-age men and women [75]. In addition, further research on information-seeking behavior as to where people could go for information and where they go for information can be helpful to identify areas of improvement [64].

The United Nations also responded to pandemics by developing guidelines and frameworks to strengthen public health response and strengthen national systems. In response to Zika, the UN created a Global Strategic Response Framework and Joint Operational Plan, which had six pillars, namely, (i) surveillance, (ii) community engagement and risk communication, (iii) vector control and personal protection, (iv) care for those affected, (v) research, and (vi) coordination. The International Medical Advisory Panel (IMAP) also prepared a statement approved by the International Planned Parenthood Federation (IPPF) for member states to ensure that sexual and reproductive health rights of women and couples are adequately protected [76]. This statement complements WHO's information and other guidelines [77–79]; in addition, WHO has published a framework designing sexual health programs that identify multisectoral approaches [80]. All these are helpful tools for national governments to adopt and utilize for developing and implementing legislation as per their needs and resources.

As this scoping review was conducted before the present pandemic COVID-19 emerged, the authors strongly believe that the lessons can also be drawn from this paper to understand and mitigate the impact of the pandemic on sexual and reproductive health services. Historically it is evident that restriction in movement and lockdown leads to confining individuals, couples, and families, leading to spending more time at home with the possibility of increased unprotected sexual activity [81]. In addition, multiple lockdowns will lead to home isolation due to the fears of contracting the virus, which can lead to a decrease in uptake of SRH services and increase in reported cases of intimate partner violence, and some geographies will reduce access to contraception and safe abortion care including in vulnerable populations and refugees [82]. It is also likely that stockouts of easily accessible short-term methods of contraception such as condoms or oral pills can happen due to global chain disruption. Thus, some estimates suggest that this pandemic will lead to higher rates of unintended pregnancy, unsafe abortion, short interpregnancy intervals, and untreated sexually transmitted infections [82].

### 4.4. Proposed Framework for Outbreaks

The World Health Organization has defined an outbreak as “a disease outbreak is the occurrence of disease cases more than normal expectancy. Several cases vary according to the disease-causing agent, and the size and type of previous and existing exposure to the agent” (https://www.who.int/environmental_health_emergencies/natural_events/en/).

Based not only on the findings emerging from this scoping review but also in the light of relevant literature on frameworks developed by a different organization such as WHO and UNFPA during various outbreaks in consultation with partners and stakeholders, the authors, therefore, are proposing a framework to identify and understand the prevailing gaps about SRH and pregnancy outcomes in the context of infectious disease-related outbreaks. It is also helpful for plugging these identified gaps with locally acceptable measures that can effectively address the needs of high-risk populations. This comprehensive framework can be used in different settings and can be modified based on locally acceptable and adoptable measures.

**Figure 3:**
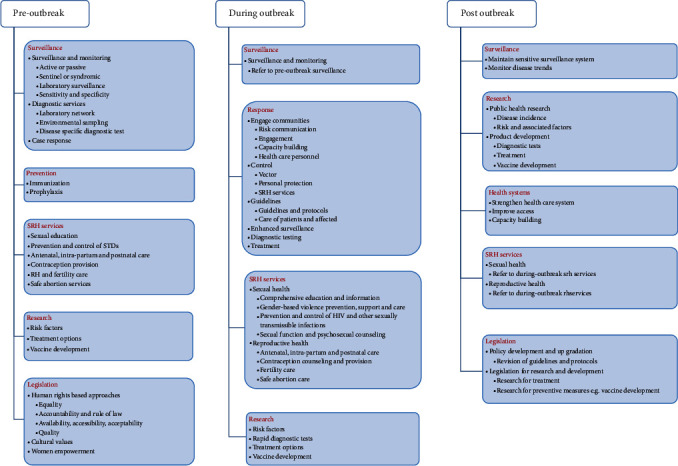
Proposed framework for outbreaks.

The proposed framework (refer to Figure 3) is divided into the following three components based on the timing of an outbreak:

1. Preoutbreak
2. During the outbreak
3. Postoutbreak

Each component deals with issues prevailing during a particular stage of an outbreak. This framework can also address prevailing gaps about any type of outbreak and is not specific to a disease. Components relevant to SRH are described here; for details on the complete framework, refer to Figure 3.

#### 4.4.1. Preoutbreak

(A) Surveillance
  (a) Surveillance and monitoring
    - Implement a sensitive surveillance system
    - Use of digital technology for communication, awareness generation, and education
  (b) Diagnostic services
    - Establish a laboratory network
  (c) Case response
(B) Sexual and reproductive health services
  - Sexual education
  - Prevention and control of HIV/STIs
  - Antenatal, intrapartum, and postnatal care
  - Contraception provision
  - Fertility care
  - Safe abortion services
(C) Legislation
  (a) Human rights-based approaches
    - Address all aspects such as equality, availability, and quality
  (b) Cultural values
  (c) Women empowerment

#### 4.4.2. During Outbreak

(A) Response
  (a) Engage communities
    - Engage communities through risk communication, engagement, and capacity building
    - Promote the use of digital technology for communication
  (b) Control
    - Vector control through PPE and improved SRH services
  (c) Guidelines
    - Develop patient care guidelines and protocols
  (d) Enhanced surveillance
  (e) Diagnostic testing
  (f) Treatment
(B) Sexual and reproductive health services

(a) Sexual health
  - Comprehensive education and information
  - Gender-based violence prevention, support, and care
  - Prevention and control of HIV and other sexually transmissible infections
  - Sexual function and psychosexual counseling
(b) Reproductive Health

- Antenatal, intrapartum, and postnatal care
- Contraception counseling and provision
- Fertility care
- Safe abortion care

#### 4.4.3. Postoutbreak

(A) Surveillance
  - Maintain a sensitive surveillance system
  - Use of digital technology for communication, awareness generation, and education
(B) Research
  - Public health research
  - Product development
(C) Legislation
  - Policy development and upgradation
  - Legislation for research and development

## 5. Conclusion

The current lack of high-quality published data related to SRH and pregnancies in an outbreak situation; the scarcity of evidence about their background in humanitarian settings, especially its accessibility; and the obstacles to using various contraceptive services raise critical concerns. Higher-quality evidence on SRH and pregnancy-related services in outbreaks are needed to improve access to SRH in outbreaks, including voluntary family planning and safe abortion services. Hence, providing greater access to rights-based equitable family planning services, addressing the use of equality could substantially contribute to achieving the SDG goals during an outbreak, especially in a global crisis at scale.

Recent outbreaks of Ebola virus disease, Zika virus disease, SARS, Middle East respiratory syndrome, and influenza H1N1 exposed high maternal morbidity and mortality rates, fetal loss, and fetal harm. As countries maintain or adjust public health measures, emergency legislation, and economic policies in response to the outbreak situations, especially the recent COVID-19 pandemic, there is an urgent need to protect the rights of and support the most vulnerable members of society. This information is essential for policymakers, donors, and program managers working within the area of SRH in outbreaks or crisis-like situations to make informed decisions regarding sustaining SRH interventions aimed at continuation of family planning use, safe abortion care, pregnancy-related services, and PAC indicators at respective national and global levels.

It is the responsibility of governments to respond at local and national levels, and the health officials are also required to provide leadership for health systems [3]. Before making any decision, the policymakers must factor in the direct effects and the indirect effects of the outbreaks. For example, the 2014 Ebola virus outbreak in West Africa showed that the indirect effects of the outbreak were more severe than the outbreak itself [2]. Based on the limited availability of dedicated frameworks on SRH and pregnancies in an outbreak situation, our review attempts to bridge the knowledge gaps through collating lessons learned derived from effective interventions to reduce adverse SRH outcomes before, during, and postoutbreaks and their implications for policymakers. The decisions taken by any government in responding to an outbreak will have consequences for the health and livelihoods of populations. In the context of these decisions, our review highlights the need to consider how to mitigate the effect of system-level disruptions and the decline in service uptake, including facility-based deliveries, contraceptive use, and antenatal and institutional care during an outbreak especially in countries with limited resources. In addition, to expedite the community- and policy-level response to the poor access issue of SRH services, the review suggested implementing gender-specific, equity-based awareness-raising interventions as key increase access and utilization of SRH services. Therefore, our analysis provides “pre-, during, and postoutbreak period” frameworks that policymakers can use to prioritize interventions and quantify the effects to inform decisions around health system continuity and the stopgap measures during and following the outbreak. In the future, it will be worthwhile to conduct a metanalysis for possible variations owing to sociodemographic indicators such as income, age, and education of the cases in the studies included in this scoping review of the available literature impacting knowledge, access, and utilization of services during a pandemic.

Outbreaks are not only public health emergencies, but they can also turn into political and socioeconomic emergencies. The lessons learned from African Ebola and cholera responses taught us to avoid the tunnel vision approach and actively address broader socioeconomic and health inequities when designing measures to counter the emergencies [83, 84]. The results will guide the next phase of a multifaceted research program that will guide further research and development of guidelines that can be implemented at the system level to measure and monitor SRH and pregnancy outcomes during outbreaks and pandemic situations.

## Figures and Tables

**Table 1:** Key definitions used in this scoping review for key concepts.

Sexual and reproductive health	Good sexual and reproductive health is a state of complete physical, mental, and social well-being in all matters relating to the reproductive system. It implies that people can have a satisfying and safe sex life, the capability to reproduce, and the freedom to decide if, when, and how often to do so. ^#^Support a life course approach to providing rights-based, accessible, quality, and integrated SRH and HIV services. As each stage in a person's life influences the next, a life course approach to SRH and HIV linkages coordinated across all stages and providers can improve delivery efficiency, uptake of services, and long-term health outcomes^^^^∗^
Source:	^#^United Nations Population Fund. Sexual & reproductive health (available from URL: https://www.unfpa.org/sexual-reproductive-health#, last accessed: Sep 09, 2018) ^^^WHO meeting on ethical, legal, and human rights and social accountability implications of self-care interventions for sexual and reproductive health: 12–14 March 2018, Brocher Foundation, Hermance, Switzerland: summary report. Geneva: World Health Organization; 2018. License: CC BY-NC-SA 3.0 IGO (available at: http://apps.who.int/iris/bitstream/handle/10665/273989/WHO-FWC-18.30-eng.pdf?ua=1) ^∗^Call to action to attain universal health coverage through linked sexual and reproductive health and rights and HIV interventions: 2018, Department of Reproductive Health and Research, World Health Organization and UNFPA (available at: http://apps.who.int/iris/bitstream/handle/10665/273148/WHO-RHR-18.13-eng.pdf)
Quality of service	For this review, we have used the WHO definition of quality of care, which is “the extent to which healthcare services provided to individuals and patient populations improve desired health outcomes. To achieve this, healthcare must be safe, effective, timely, efficient, equitable, and people-centered [18, 19].” (i) *Safe*. It delivers healthcare that minimizes risks and harm to service users, including avoiding preventable injuries and reducing medical errors (ii) *Effective*. We are providing services based on scientific knowledge and evidence-based guidelines (iii) *Timely*. We are reducing delays in providing and receiving healthcare (iv) *Efficient*. We are delivering healthcare in a manner that maximizes resource use and avoids waste (v) *Equitable*. It delivers healthcare that does not differ in quality according to personal characteristics such as gender, race, ethnicity, geographical location, or socioeconomic status (vi) *People-centered*. It is providing care that considers the preferences and aspirations of individual service users and the culture of their community
Access	The availability of good health services within reasonable reach of those who need them and opening hours, appointment systems, and other aspects of service organization and delivery allow people to obtain the services when they need them [20]
	As defined in the human rights context, “health facilities, goods, and services must be within safe physical reach for all sections of the population, especially vulnerable or marginalized groups, such as ethnic minorities and indigenous populations, women, children, adolescents, older persons, persons with disabilities and persons with HIV/AIDS, including in rural areas”
Health-seeking behavior	Health-seeking behavior is any action carried out by a person who perceives a need for health services to address a given health problem. This includes seeking help from allopathic and alternative health services, and both sex and gender influence health-seeking behavior [21]
Pregnancy outcomes	A pregnancy outcome is the result of a fertilization event. Types of pregnancy outcomes include miscarriage, live birth (full-term or preterm birth), stillbirth, spontaneous abortion, and induced abortion [22]
Definitions of pregnancy outcomes	
*Miscarriage*	Miscarriage is defined as the spontaneous loss of pregnancy before the fetus reaches viability. The term, therefore, includes all pregnancy losses from the time of conception until 24 weeks of gestation (RCOG Green-top Guideline No. 17. https://www.rcog.org.uk/globalassets/documents/guidelines/gtg_17.pdf)
*Stillbirth*	A baby born with no signs of life at or after 28 weeks' gestation
*Low birthweight*	A baby born weighing <2500 g regardless of gestation
*Neonatal death*	A baby born alive but who dies within the first 28 days of life

**Table 2:** Frequency of records mentioning major outbreak causing microbial organisms.

	*n*	%
Chikungunya	90	2.54
Cholera	229	6.46
Crimean-Congo hemorrhagic fever	1	0.03
Ebola virus disease	554	15.62
Hendra virus infection	7	0.20
Influenza (pandemic, seasonal, and zoonotic)	1334	37.61
Lassa fever	10	0.28
Marburg virus disease	25	0.70
Meningitis	319	8.99
MERS-CoV	13	0.37
Monkeypox	15	0.42
Nipah virus infection	2	0.06
Plague	91	2.57
Rift Valley fever	28	0.79
SARS	240	6.77
Smallpox	123	3.47
Tularemia	2	0.06
Yellow fever	81	2.28
Zika virus disease	383	10.80

**Table 3:** Descriptive characteristics of the included studies.

Sr#	Study ID	Year	Country/area	Settings	Scope	Study design	Outbreak	Health aspect
1	Adams et al.	2016	Puerto Rico	Urban	Subnational	Prospective	Zika	Pregnancy
2	Ali et al.	2018	Brazil	Both	National	Retrospective	Zika	SRH
3	Ali et al.	2018	Brazil/City of Santana do Ipanema, Alagoas, City of Balsas and Sao Luis, Maranhao		Subnational	Mixed methods—cross-sectional, interviews	Zika	SRH
4	Ali et al.	2018	Brazil	Both	National	Prospective/retrospective	Zika	SRH
5	Roa et al.	2016	Latin America	Both	Regional	Commentary	Zika	SRH
6	Bahamondes et al.	2017	Brazil	Both	National	Not reported	Zika	SRH
7	Bebell et al.	2017	Africa	Both	Regional	Cross-sectional	Ebola	Pregnancy outcomes
8	Besnard et al.	2016	French Polynesia	Both	National	Retrospective	Zika	Pregnancy outcomes
9	Borges et al.	2018	Brazil	Urban	Subnational	Cross-sectional	Zika	SRH
10	Camara et al.	2017	Guinea	Both	National	Prospective/Retrospective	Ebola	SRH
11	Castro et al.	2018	Brazil	Both	National	Prospective	Zika	Pregnancy outcomes
12	Ciglenecki et al.	2013	Haiti	Urban	Subnational	Prospective	Cholera	Pregnancy
13	da Sliva et al.	2014	Brazil	Urban	Subnational	Prospective	H1N1	Pregnancy outcomes
14	Delamou et al.	2017	Guinea	Rural	Subnational	Retrospective	Ebola	Pregnancy outcomes
15	Flamand et al.	2017	Guiana	Both	National	Retrospective	Zika	Pregnancy outcomes
16	Fonseca et al.	2016	Brazil	Urban	Subnational	Prospective	Zika	Pregnancy outcomes
17	Garde et al.	2016	Sierra Leone	Urban	Subnational	Retrospective	Ebola	Pregnancy
18	Guetiya et al.	2017	Sierra Leone	Rural	Subnational	Retrospective	Ebola	SRH
19	Gunaratne et al.	2006	Sri Lanka	Rural	Subnational	Retrospective	Meningitis	SRH
20	Henwood et al.	2017	Liberia & Sierra Leone	Urban	Subnational	Retrospective	Ebola	Pregnancy outcomes
21	Hyjazi et al.	2015	Guinea	—	—	Retrospective	Ebola	SRH
22	Jones et al.	2017	Sierra Leone	Both	National	Prospective	Ebola	Both
23	Journel et al.	2017	Haiti	Both	National	Prospective	Zika	Pregnancy outcomes
24	Kallam et al.	2017	Guatemala, El Salvador, Dominican Republic, Honduras, Haiti	—	—	Prospective	Zika	Pregnancy outcomes
25	Kanmaz et al.	2011	Turkey	Urban	Subnational	Prospective	Influenza A	Pregnancy outcomes
26	Lathrop et al.	2018	Puerto Rico	Urban	Subnational	Prospective	Zika	SRH
27	Leno et al.	2018	Guinea	Both	—	Retrospective	Ebola	SRH
28	Lori et al.	2015	Liberia	Rural	Subnational	Retrospective	Ebola	SRH
29	Luginaah et al.	2016	Liberia	Both	National	Retrospective	Ebola	SRH
30	Ly et al.	2016	Liberia	—	Subnational	Prospective	Ebola	SRH
31	Marteleto et al.	2017	Brazil	—	Subnational	Prospective	Zika	SRH
32	Mupapa et al.	1999	Congo	Rural	Subnational	Retrospective	Ebola	Pregnancy outcomes
33	Tavares et al.	2016	Brazil	Urban	Subnational	Cross-sectional	Zika	SRH
34	Tepper et al.	2016	Puerto Rico	Both	National	Cross-sectional	Zika	SRH
35	Borlin et al.	2016	Sierra Leone	Both	National	Prospective	Ebola	SRH
36	Theuring et al.	2018	Sierra Leone	Urban	Subnational	Focus group discussion	Ebola	Pregnancy outcomes
37	Miller et al.	2018	Guinea, Liberia, and Sierra Leone	Both	Subnational	Mixed methods	Ebola	SRH

**Table 4:** Study objectives and key findings.

Sr#	Study ID	Year	Objective	Outcome measure	Key findings 1	Key findings 2	Key findings 3
1	Adams et al.	2016	Burden of disease	Incidence of Zika virus case in Puerto Ricco	Zika virus epidemic is gaining momentum	Need vector control and personal protection equipment	
2	Ali et al.	2018		Contraceptive sales	Little variation during Zika outbreak;	Very low access to LARC methods	
3	Ali et al.	2018	Health system readiness	The facility records for contraceptive stock availability, methods mix offered, and utilization pattern; the facility managers and senior health officials in the state were also interviewed	Shortage and stockouts of both short- and long-term contraceptives and long-acting reversible contraceptives (LARCs) were either absent or mostly out of stock	None of the facilities surveyed had either a national abortion guideline or safe abortion check lists	
4	Ali et al.	2018	Contraceptive use	Sales of various contraceptive products	The results from this assessment showed that the sales of contraceptives presented little variation during the ZIKV outbreak in Brazil		
5	Rao	2016			Lack of awareness, poverty	Sex education and access to contraceptives, safe motherhood, safe abortion, and programs to prevent discrimination and exclusion of people living with disabilities	
6	Bahamondes et al.	2017	Contraceptive use	Sales of various contraceptive products	No significant change in contraceptive sales		
7	Bebell et al.	2017	Update on Ebola	Mental mortality	Improved survival with time		
8	Besnard et al.	2016	Report on pregnancy outcomes	Pregnancy outcomes	Medical Abortion in 11/19 cases, infant death in 2/19 cases, neurological impairment in 6/19 cases		
9	Borges et al.	2018	KAP	Pregnancy intentions, contraceptive practice knowledge	Awareness is relatively low, few women changing pregnancy intentions		
10	Camara et al.	2017	SRH & healthcare utilization	Contraceptive, antenatal care use, and institutional deliveries	All services affected by Ebola. Decline in all three types of services	Contraceptive services recovered postoutbreak; ANC and institutional deliveries did not	
11	Castro et al.	2018	Assessment of decline in number of live births	Liver births, Zika virus syndrome	Observed and forecasted number of live births		
12	Ciglenecki et al.	2013	Documenting outcomes in pregnant women with Cholera	Pregnancy outcomes	86% preserved pregnancy, 8% fetal death, 6% live birth		
13	da et al.	2014	Documenting outcomes in pregnant women exposed to H1N1	Pregnancy outcomes	Higher health service consumption and deaths observed as compared to non-HIN1 influenza cases and influenza-negative patients		
14	Delamou et al.	2017	Effect of Ebola on MNCH service delivery	Eight MNCH health service indicators	Health service utilization indicators worsened during outbreak. Worsening improved postoutbreak but did not indicate recovery		
15	Flamand et al.	2017	Asymptomatic cases burden	Asymptomatic cases of Zika	A considerable proportion of women are asymptomatic		
16	Fonseca et al.	2016	Describe healthcare provision during double epidemic in ER	Burden of disease	DF disrupted routine care		
17	Garde et al.	2016	Describe healthcare provision using triage and isolation	EVD disease protocol implementation	Increased efficiency and quality were seen in patient flow, screening accuracy, nursing skills, IPC, and quality of care	This model can be followed at other places for better service provision	
18	Guetiya et al.	2017	Implementation of mobile health clinics	Utilization of mobile clinics	Mobile health clinics were received enthusiastically by the communities	Mobile health clinics address many barriers related to uptake of services among EVD survivors, particularly in remote areas	
19	Gunaratne et al.	2006	Outbreak investigation		Suboptimal storage conditions for medical supplies (regular & donated posttsunami)	In availability of optimal storage space for donated medical supplies	
20	Henwood et al.	2017	Report on pregnancy outcomes	Maternal and neonatal deaths	Maternal deaths are not different from non-Ebola-infected mothers	Neonatal outcomes are poor suggested by limited data	
21	Hyjazi et al.	2015	Report on healthcare service utilization	Utilization of institutional care	The results from this assessment showed that the healthcare utilization reduced greatly due to outbreak		
22	Jones et al.	2017	Role of nurses/midwives in providing healthcare during Ebola outbreak	(Qualitative study)	Nurses and midwives faced higher risk of catching Ebola compared to their health workers but continued to provide essential maternity care	Due to profession duty, responsibility to community, and religious beliefs	
23	Journel et al.	2017	Implementation of surveillance system	Burden of disease	Implementation of monitoring of Zika virus disease including labs, vector control, social mobilization, and clinical care		
24	Kallam et al.	2017	Upgradation of ultrasonographic capacity	Care of equipment, ultrasound provided capacity, service delivery observation, and client volume referral pattern	Ongoing data collection	Need upgradation of diagnostic services	
25	Kanmaz et al.	2011	Strategies to handle patient load during influenza A outbreak	Patient handling strategy	Reduction of adverse outcomes likely due to strategic handling of pregnant women		
26	Lathrop et al.	2018	Providing free-of-cost reversible contraception to women through a network of providers	Increased utilization of contraception by women	Increased adoption of most effective methods, reduction in nonusers, and users of least effective methods		
27	Leno et al.	2018	Healthcare service utilization	Antenatal care visits	Reduction of utilization of healthcare services during Ebola outbreak in centers located in affected areas but not in unaffected areas of Guinea		
28	Lori et al.	2015	Healthcare service utilization	Facility-based deliveries	EVD adversely affected rising trend of facility-based deliveries in the Bong county		
29	Luginaah et al.	2016	Healthcare service utilization	Timing of first ANC visit	Women delayed first ANC due to stigma attached to the facility		
30	Ly et al.	2016	Healthcare service utilization	Facility-based deliveries	Facility-based deliveries reduced	Reduction was significant among those who believed the facility to be a risk factor for contracting Ebola	
31	Marteleto et al.	2017	Reproductive intentions and behavior of women during Zika outbreak	—	Most women intend to delay pregnancy	High-SES women had more control on their choices as compared to low-SES women	
32	Mupapa et al.	1999	Mortality in EHF mothers admitted to a hospital	Pregnancy outcomes	Very high mortality in mothers admitted with EHF in Kiewit, DRC		
33	Tavares et al.	2016	Availability of emergency contraception in northern urban Brazil	Availability of EC at pharmacy	Almost all of the pharmacies have EC available	The knowledge of pharmacist need to be improved	
34	Tepper et al.	2016	Contraceptive needs and access to contraception	Unmet need	There is large gap between need and availability of contraception in Puerto Rico	In the backdrop of a large number of unintended pregnancies, there is a need to improve availability and access to contraception	
35	Borlin et al.	2016	Impact of outbreak on institutional deliveries & C-sections	Institutional deliveries & C-sections	Outbreak broke the upward trend of higher utilization of SRH in Sierra Leone		
36	Theuring et al.	2018	External and intrinsic barriers to facility-based delivery	Facility-based delivery	More supportive staff attitudes; acceptance of an accompanying person throughout delivery	Better referral system and ambulance services	
37	Miller et at.	2018	Impact of Ebola on community-based maternal, newborn, and child health (MNCH) services	MNCH services	Sharp decline in MNCH services due to weak service delivery, confusion over policy, and overwhelming nature of outbreak	Services rebounded when clear instructions were given. Despite mistrust, CHWs were more effective than outsiders	Service delivery weaknesses, especially related to supply chain and supervision, limited the effectiveness of community health services before, during, and after the outbreak

## Data Availability

It is a scoping review type of a review article where peer reviewed journal articles were used in the literature review are available from open sources such as MEDLINE, EMBASE and ISI-WoS.

## Appendix

### A.1. MEDLINE Search Strategy (as of Jul 3, 2018)

1. exp CHIKUNGUNYA FEVER/or Chikungunya.mp.
2. Cholera.mp. orexp CHOLERA/
3. exp Hemorrhagic Fever, Crimean/or Crimean-Congo hemorrhagic fever.mp.
4. Hendra virus infection.mp. OrexpHenipavirus Infections/
5. Influenza.mp. orexp Influenza, Human/
6. Lassa fever.mp. orexp Lassa Fever/
7. Marburg virus disease.mp. orexp Marburg Virus Disease/
8. Meningitis.mp. orexp MENINGITIS/
9. MERS-CoV.mp. orexp Middle East Respiratory Syndrome Coronavirus/
10. exp MONKEYPOX/or Monkeypox.mp.
11. Nipah virus infection.mp. orexpHenipavirus Infections/
12. exp PLAGUE/ or Plague.mp.
13. Rift Valley fever.mp. orexp Rift Valley Fever/
14. exp severe acute respiratory syndrome/or SARS.mp.
15. Smallpox.mp. orexp SMALLPOX/
16. exp Tularemia/or Tularaemia.mp.
17. Yellow fever.mp. orexp Yellow Fever/
18. Yellow fever.mp. orexp Yellow Fever/
19. expZika Virus Infection/ or expZika Virus/or zika.mp.
20. exp Ebolavirus/ or exp Hemorrhagic Fever, Ebola/or Ebola.mp.
21. 1 or 2 or 3 or 4 or 5 or 6 or 7 or 8 or 9 or 10 or 11 or 12 or 13 or 14 or 15 or 16 or 17 or 18 or 19 or 20
22. birth control.mp. orexp Contraception/
23. family planning.mp. orexp Family Planning Services/
24. Contraceptive services.mp.
25. exp Abortion, Induced/ or exp Abortion, Spontaneous/or abortion.mp.
26. pregnancy termination.mp.
27. exp Cesarean Section/or c-section.mp. or c section.mp. or cesarean.mp.
28. prenatal.mp. orexp Prenatal Care/or antenatal.mp.
29. pregnancy complications.mp. orexp pregnancy Complications/
30. delivery.mp. orexp Delivery, Obstetric/
31. (post-natal or postnatal or post-natal or ((post or post-) and care)).mp.
32. (post-abortion care or post-abortion care or postabortion care).mp.
33. exp ∗Reproductive Health Services/or Reproductive health service.mp.
34. or/22-33
35. epx ∗ disease outbreaks/or epidemic∗.mp.
36. 21 and 34 and 35
37. limit 36 to ed=19950101-20180531

### A.2. Embase Search Strategy (as of Jul 3, 2018)

A closely matching search strategy was run for Embase with exact date limits.

### A.3. ISI Web of Science

Diseases: CHIKUNGUNYA FEVER or Cholera or Hemorrhagic Fever or Crimean or Hendra virus infection or Influenza or Lassa fever or Marburg virus disease or Meningitis or MERS-CoV or MONKEYPOX or Nipah virus infection or PLAGUE or Rift Valley fever or severe acute respiratory syndrome or Smallpox or Tularemia or Yellow fever or yellow fever or Zika Virus Infection or Ebolavirus

Reproductive health services: birth control or Contraception or family planning or Family Planning Services or Contraceptive services or Abortion, Induced or Abortion, Spontaneous or abortion or pregnancy termination or Cesarean Section or c-section or c section or cesarean or prenatal or Prenatal Care or antenatal or pregnancy complications or Pregnancy Complications or delivery or Delivery, Obstetric or (post-natal or post-natal or post-natal or ((post or post-) and care)) or (post-abortion care or post-abortion care or post-abortion care) or Reproductive Health Services or Reproductive health service

Epidemic: Disease outbreak or Epidemic

Date limit: Jan 01, 1995, to Jul 3, 2018 (as no more precise date restriction applicable)
